# Pre-clinical blocking of PD-L1 molecule, which expression is down regulated by NF-κB, JAK1/JAK2 and BTK inhibitors, induces regression of activated B-cell lymphoma

**DOI:** 10.1186/s12964-019-0391-x

**Published:** 2019-08-05

**Authors:** Christelle Vincent-Fabert, Lilian Roland, Ursula Zimber-Strobl, Jean Feuillard, Nathalie Faumont

**Affiliations:** 1 UMR-CNRS 7276/INSERM U1262 CRIBL “Contrôle de la Réponse Immune B et Lymphoproliférations”, CBRS “Centre de Biologie et de Recherche en Santé”, Dupuytren Hospital University Center, University of Limoges, Hematology Laboratory of Dupuytren CHU, 2 rue du Pr Descottes, 87025 Limoges, France; 2 Research Unit Gene Vectors, Helmholtz Center Munich, German Research Center for Environmental Health GmbH, Munich, Germany

**Keywords:** B-cell lymphomas, PD-L1, Immune surveillance

## Abstract

Escape from immune control must be important in the natural course of B-cell lymphomas, especially for those with activation of NF-κB. The pre-clinical LMP1/CD40-expressing transgenic mouse model is characterized by B-cell specific CD40 signaling responsible for NF-κB continuous activation with a spleen monoclonal B-cell tumor after 1 year in 60% of cases. LMP1/CD40 tumors B-cells expressed high levels of PD-L1. This expression was dependent on activation of either NF-κB, JAK1/JAK2 or BTK pathways since these pathways were activated in tumor B-cells and ex vivo treatment with the inhibitory molecules PHA-408, ruxolitinib and ibrutinib led to decrease of its expression. Treatment of LMP1/CD40-expressing lymphomatous mice with an anti-PD-L1 monoclonal antibody induced tumor regression with decreased spleen content, activation and proliferation rate of B-cells as well as a marked increase in T-cell activation, as assessed by CD62L and CD44 expression. These results highlight the interest of therapies targeting the PD-1/PD-L1 axis in activated lymphomas with PD-L1 expression, with possible synergies with tyrosine kinase inhibitors.

**Electronic supplementary material:**

The online version of this article (10.1186/s12964-019-0391-x) contains supplementary material, which is available to authorized users.

## Background

Aberrant expression of the programmed death-ligand 1 (PD-L1, also known as B7-H1 or CD274) checkpoint molecule has been reported in many cancers such as breast, lung and colon tumors as well as during chronic viral infections like those with Epstein-Barr virus (EBV) for example [1, 2]. Efficacy of immunotherapies against the PD-1/PD-L1 axis in lung tumors or melanomal demonstrated the importance of the immune checkpoints in the control of emergence and growth of tumors [2]. As reviewed recently, various publications have indicated that disruption of immune checkpoints is also a critical step in B-cell non-Hodgkin’s Lymphomas (NHL) [3]. NF-κB, one of the most cited transcription factor in B-cell lymphomas, is able to increase tumor cell expression of PD-L1 either directly or indirectly [3]. NF-κB constitutive activation is found either in aggressive diffuse large B-cell lymphomas (DLBCL) with an activated phenotype (ABC-DLBCL), or in indolent B-cell lymphomas such as chronic lymphocytic leukemia, Waldenström Macroglobulinemia, marginal zone B-cell lymphomas (MZL) [4]. Here, we wanted to explore the putative interest of PD-L1 immune therapy against B-cell lymphoma with NF-κB activation. To experimentally address this question, we used a transgenic mouse model which specifically express in B-cells a chimeric protein composed of the transmembrane moiety of the Epstein-Barr Virus latent membrane protein 1 (LMP1) and the transduction tail of CD40 (LMP1/CD40 protein), that results in continuous activation of NF-κB, responsible for a spleen monoclonal B-cell tumor (LMP1/CD40 B-cell lymphoma) after 1 year in 60% of cases [5].

## Methods

### Mouse models and in vivo and ex vivo treatments

LMP1/CD40-expressing mice have been already described [5]. Animals were housed at 21–23 °C with a 12-h light/dark cycle. All procedures were conducted under an approved protocol according to European guidelines for animal experimentation (French national authorization number: 87–022 and French ethics committee registration number “CREEAL”: 09-07-2012). For in vivo PD-L1 treatment, LMP1/CD40-expressing mice were injected intraperitoneally every 4 days for 3 weeks with 200 μg anti-PD-L1 antibody (clone 10F.9G2; Bio X cell; US). For ex vivo treatments, splenocytes were cultured for 48 h in complete RPMI medium (Eurobio) supplemented with 10% of FBS, 2 mM of L-Glutamine, 1% of Na pyruvate, 100 U/ml of penicillin and 100 μg/ml of streptomycin (ThermoFisher Scientific) and with the following treatments: either 10 μM of PHA-408 or 1.5 μM of ruxolitinib or 1 μM of ibrutinib.

### Flow cytometry

Spleen from mice were collected and immune cells were filtered through a sterile nylon membrane. Cell suspensions were stained at 4 °C in FACS Buffer (PBS, 1% FBS, 2 mM EDTA) with the following fluorescent-labelled antibodies: anti B220-BV421 (clone RA3-6B2, 1/400), anti CD4-FITC (clone RM4–5, 1/2000), anti CD8a-APC (clone 53–6.7, 1/400), anti CD62L-BV421 (clone MEL-14, 1/200), anti CD44-PE (clone IM7, 1/200), anti-PD-L1-PE (clone 10F.9G2, 1/80), anti PD-1-PECy7 (clone 29F.1A12, 1/50), anti CD80-APC (clone16-10A1, 1/2500) and anti CD86-FITC (clone GL-1, 1/600). Stained cells were analyzed using a BD-Fortessa SORP flow cytometer (BD Bioscience; US). Results were analyzed using Kaluza Flow Cytometry software 1.2 (Beckman Coulter; France).

### Proliferation

For in vivo proliferation, mice were injected intraperitoneally with 2 mg BrdU (Sigma-Aldrich, US), 18 h before isolating cells. Splenocytes were stained for B220 and phases of cell cycle were analyzed by measuring BrdU and Propidium Iodide (PI)-incorporation, using the FITC-BrdU Flow Kit (BD Pharmingen; US).

### Real time quantitative reverse-transcription PCR

Total RNA was extracted from CD19_Cre or LMP1/CD40 splenocytes and complementary DNAs were reverse transcribed from 1 μg of total RNA samples using the High Capacity cDNA Archive Kit (Life Technologies, Carlsbad, USA). PCR products were amplified from each cDNA using the TaqMan® Universal PCR Master Mix and TaqMan® Gene Expression Assays: IL-10/Mm01288386_m1, PD-L1/Mm03048248_m1, GAPDH/Mm99999915_g1. The GAPDH gene was used as a reference gene for the control of amplification. The calculated relative gene expression level was equal to 2 − DDCT, where DDCT is the delta delta cycle threshold. Gene expression fold changes were calculated as the ratio of the test condition to its control.

### Immunoblot

10 × 10^6^ cells were lysed and sonicated in Laemmli sample buffer. Lysates were separated on 12% SDS-PAGE gel and transferred to PVDF membrane. Revelation were done using anti-TRAF1 antibody (N-19) purchased from Santa Cruz Biotechnology; anti-STAT3 antibody (124H6) and anti-phosphoSTAT3 antibody (Tyr 705) (D3A7) from Cell Signaling Technology; and anti-ERK (p-44/42) antibody and anti-phosphoERK antibody (Thr 202/Tyr 204) from Cell Signaling Technology. Signals were visualized with Immobilon™ Western Chemiluminescent HRP Substrate (Millipore). The amount of loaded protein was standardized against GAPDH using an anti-GAPDH antibody from R&D Systems. Membranes were numerized with ChemiDoc™ Touch Imaging System (Bio-Rad) and images were analysed with the Image Lab Software (Bio-Rad).

## Results

**Fig. 1:**
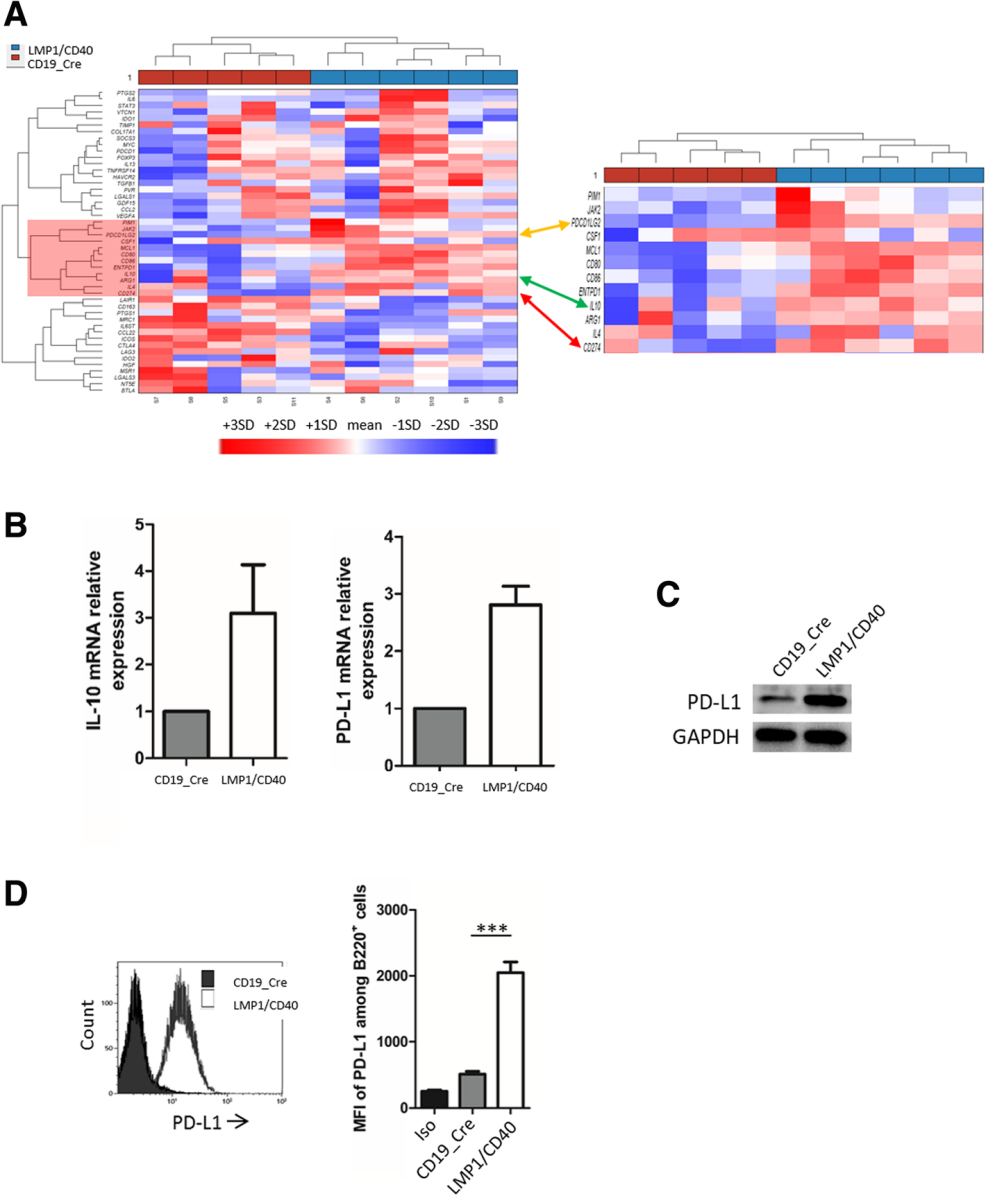
**a** Clustering of genes from the Immune Escape Gene Signature published by C Laurent et al. for LMP1/CD40 and CD19_Cre mice. The specific cluster for LMP1/CD40-expressing mice is highlighted in red. PD-L1 is pointed by the arrow. **b** Analysis of IL-10 and PD-L1 expression by RT-QPCR and (**c**) western-blot analysis of PD-L1 expression from 12-month-old CD19_Cre and LMP1/CD40-expressing mice. **d** Left panel, overlay example of PD-L1 monoparametric histograms gated on B220 B-cells. Right panel, flow cytometry Mean Fluorescence Intensity (MFI) of PD-L1 on B-cells from 12-month-old control CD19_Cre and transgenic LMP1/CD40-expressing mice. Labelling with isotype antibody (Iso). Statistical significance was determined by unpaired t-test (***P < 0.001)

Our previous transcriptome studies from LMP1/CD40-expressing mice suggested that those tumors might express high levels of CD274/PD-L1 [6]. We thus analyzed the Immune Escape Gene Signature published by C Laurent et al (Additional file 1: Table S1) [7] from the Affymetrix transcriptome (HT MG-430 PM Array, Additional file 1: Table S2 and Additional file 2: Table S3) of a series of six LMP1/CD40 B-cell lymphomas that were compared to their CD19_Cre littermate. As shown in Fig. 1a, both PD-L1 (red arrow) and PD-L2 (orange arrow) were co-clusterized with the immunosuppressive interleukin 10 (IL-10, green arrow), CD80 and MCL1, being over expressed. We confirmed overexpression of IL-10 and PD-L1 by RT-QPCR in 12-month-old LMP1/CD40-expressing mice when compared to CD19_Cre mice (Fig. 1b). We also showed higher levels of total PD-L1 expression by immunoblotting and of PD-L1 exported to the membrane by flow cytometry on LMP1/CD40 lymphoma cells from those mice than on B-cells from age related CD19_Cre mice (Fig. 1c and d).

**Fig. 2:**
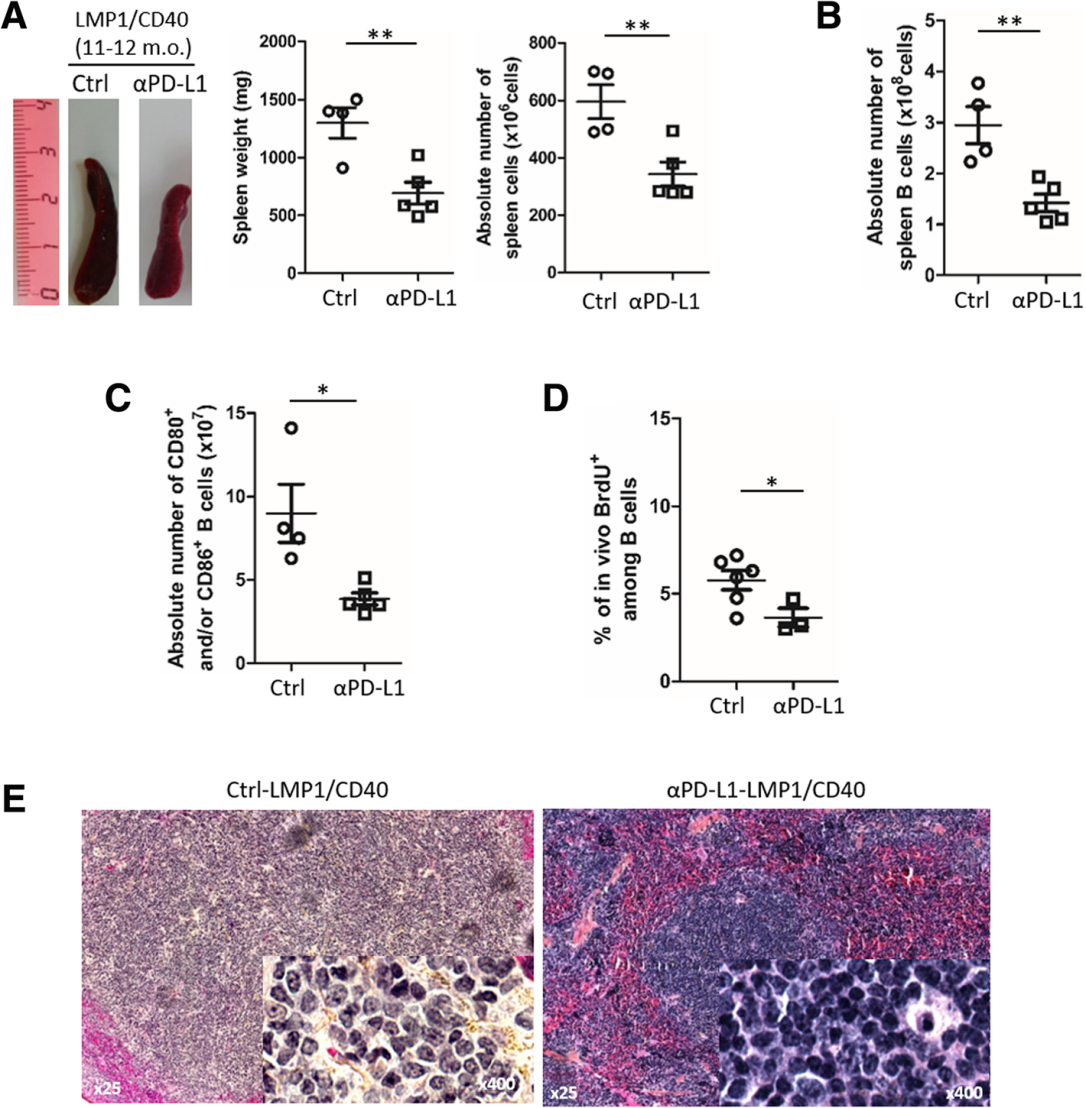
**a** Left panel, examples of whole spleens from isotype control (Ctrl) and anti-PD-L1 (αPD-L1) antibody treated LMP1/CD40-expressing mice; middle panel, mean and standard deviation of spleen weight; right panel, absolute numbers of spleen B cells. For the PD-L1 treatment, LMP1/CD40-expressing mice were injected every 4 days for three weeks with 200 μg anti-PD-L1 antibody in In VivoPure Dilution Buffer (clone 10F.9G2; Bio X cell; US). **b** Spleen B220 B-cell absolute numbers assessed by flow cytometry in Ctrl and αPD-L1 treated LMP1/CD40-expressing mice. **c** Absolute numbers assessed by flow cytometry of spleen B220 B-cells expressing CD80 and/or CD86 activation markers in LMP1/CD40-expressing mice after injection of isotope control (Ctrl) or anti-PD-L1 (αPD-L1) antibody. **d** Mean and standard deviation of flow cytometry percentages of BrdU positive B-cells after in vivo BrdU incorporation in Ctrl and αPD-L1 treated LMP1/CD40-expressing mice. Mice were injected intraperitoneally with 2 mg BrdU, 18 h before isolating cells. (**e**) Hematein eosin staining of 5 μm section of paraffin embedded spleen tissues with, as insert, the imprint May-Grunwald staining of the same spleen of Ctrl- (left panel) and αPD-L1- (right panel) LMP1/CD40-expressing mice. Statistical significance was determined by unpaired t-test (**P < 0.01; *P < 0.05)

**Fig. 3:**
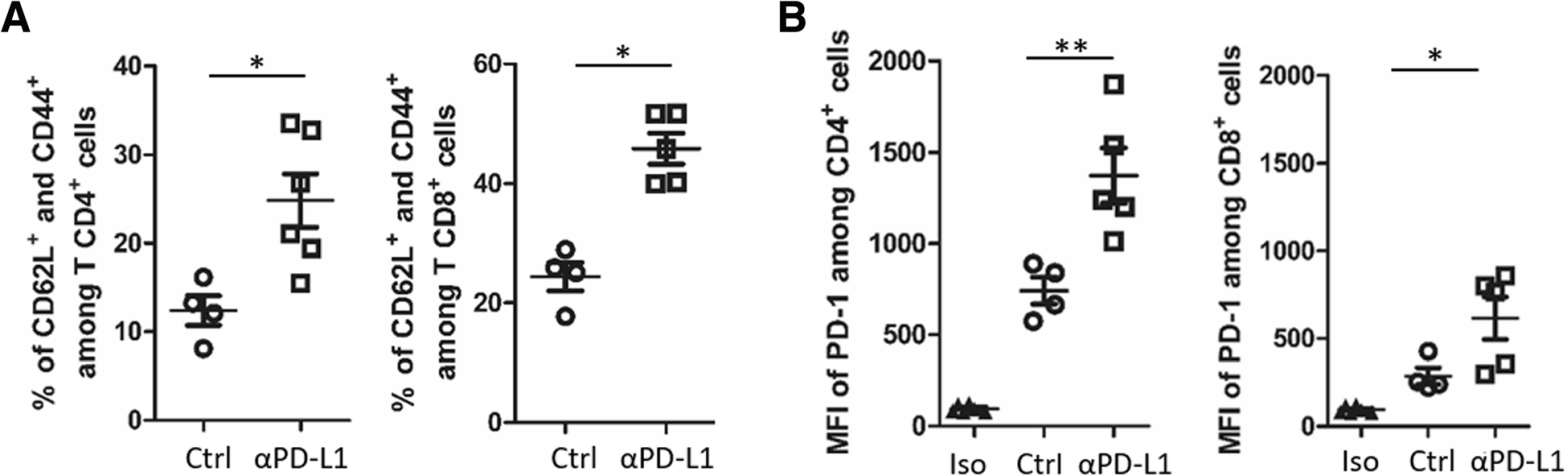
**a** Mean and standard deviation of flow cytometry percentages of activated CD4 and CD8 T-cells expressing CD62L and CD44 activation markers in LMP1/CD40-expressing mice after injection of isotype control (Ctrl) or anti-PD-L1 (αPD-L1) antibody. **b** Flow cytometry Mean Fluorescence Intensity (MFI) of PD-1 on CD4 and CD8 T-cells in Ctrl and αPD-L1 treated LMP1/CD40-expressing mice. Statistical significance was determined by unpaired t-test (**P < 0.01; *P < 0.05)

To address in vivo the role of PD-L1 in these B-cell lymphoma, LMP1/CD40-expressing mice were treated with an antibody blocking PD-1/PD-L1 signaling for 3 weeks according to a methodology already described [8, 9]. We observed a reduction of spleen size and absolute number of splenocytes in anti-PD-L1 treated LMP1/CD40-expressing mice (Fig. 2a), due to decreased B-cell numbers (Fig. 2b) without significantly affecting total numbers of T-cells and granulocytes (Additional file 3: Figure S1). With PD-L1 blockade, we noticed a decrease in numbers of activated B-cells in LMP1/CD40-expressing mice (Fig. 2c). This was associated with a reduction in the in vivo proliferation rate of spleen B-cells, as assessed by the decrease of in vivo BrdU incorporation over 18 h (Fig. 2d). Morphologically, spleen lymphocytes from anti-PD-L1 treated LMP1/CD40-expressing mice were smaller with a more condensed chromatin (Fig. 2e). We then studied the impact of PD-1/PD-L1 blockade in T-cell compartment. Expression of T-cell activation markers CD62L and CD44 was increased on both CD4 and CD8 T-cells (Fig. 3a). In parallel, an increase in PD-1 expression was observed on the surface of CD4 T-cells. This increase in PD-1 expression was more heterogeneous on CD8 T-cells (Fig. 3b).

**Fig. 4:**
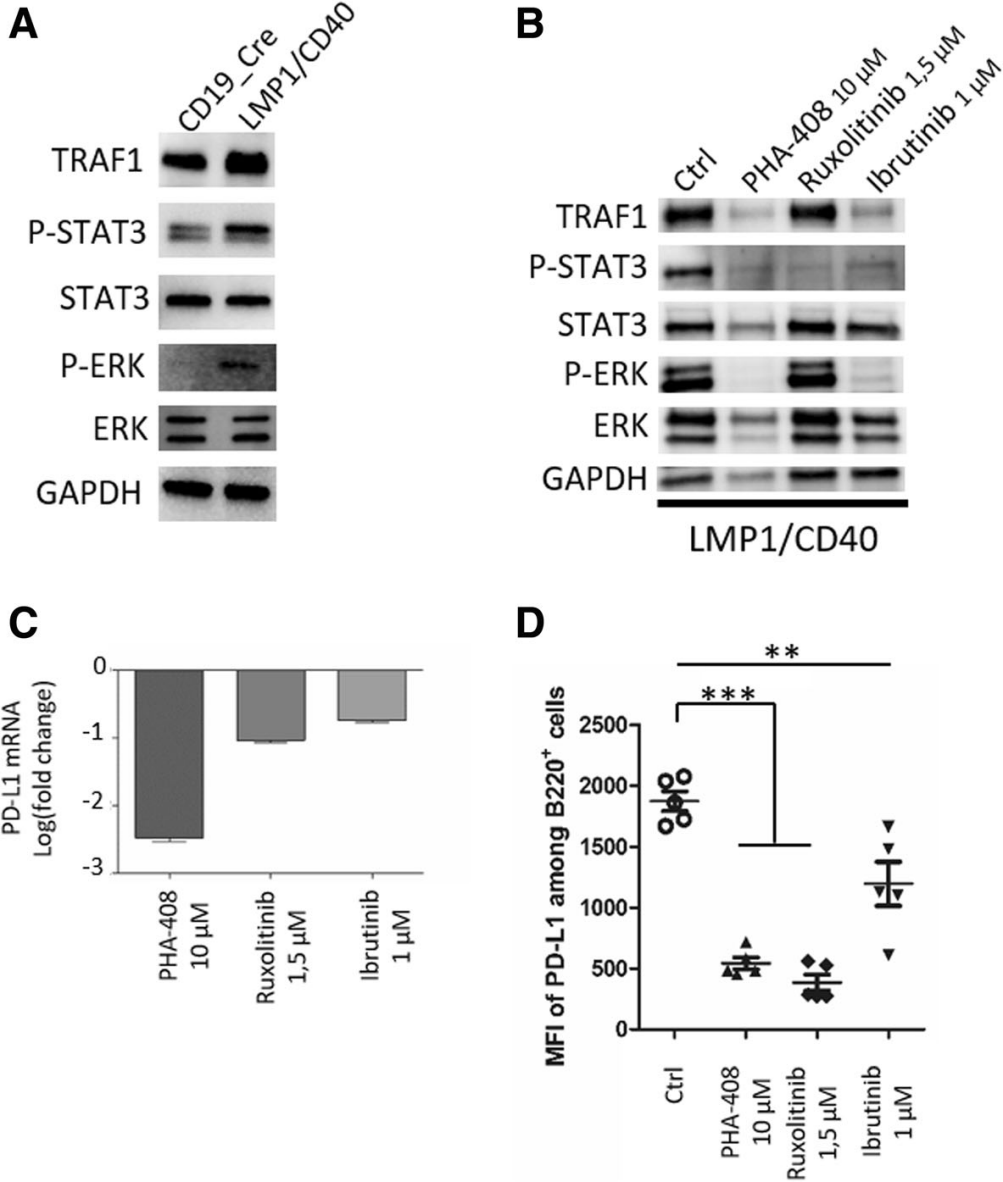
Analysis of NF-κB/TRAF1, JAK/STAT3, and ERK pathways by western blot from protein extracts of (**a**) splenocytes from control CD19_Cre and transgenic LMP1/CD40-expressing mice; (**b**) splenocytes from LMP1/CD40-expressing mice after 48 h in vitro inhibitor treatments (PHA-408, Ruxolitinib and Ibrutinib). GAPDH was used as loading control. **c** PD-L1 mRNA expression in splenocytes from LMP1/CD40-expressing mice treated by inhibitors (PHA-408, Ruxolitinib and Ibrutinib) compare to untreated cells. Data were expressed in logarithm of fold change. **d** Flow cytometry Mean Fluorescence Intensity (MFI) of PD-L1 on gated B220+ B-cells after 48 h in vitro inhibitor treatments (PHA-408, ruxolitinib and ibrutinib). Statistical significance was determined by unpaired t-test (***P < 0.001; **P < 0.01)

In addition to be able to activate the classical pathway, CD40 is a strong inducer of the alternative NF-κΒ activating pathway as already shown in LMP/CD40 mice [5]. CD40 constitutive activation of B-cells increases IL-10 expression [10]. IL-10 receptor signaling is mediated by the JAK1 and Tyk2 tyrosine kinase that leads to activation of STAT3 transcription factor, STAT3 being a major inducer of PD-L1 gene expression [11]. CD40 stimulation can also augment BCR-induced B cell responses by activation of Bruton’s tyrosine kinase (BTK) either directly [12] or by interacting with CD19 [13]. The BCR is also able to induce the IL-10/STAT3 signaling with increased expression of PD-L1 in diffuse large B-cell lymphoma [11]. Comparing splenocytes of LMP1/CD40 mice to those of CD19_Cre mice, allowed to check activation of NF-κB, as assessed by increased expression of TRAF1. Increased phosphorylation of STAT3 and ERK indicated that IL-10 and BCR-related signaling pathways were also constitutively activated (Fig. 4a). LMP1/CD40 lymphoma B-cells were ex vivo treated with the PHA-408 molecule, an inhibitor of IKK2/NF-κB activation, the JAK1/JAK2 tyrosine kinase inhibitor (TKI) ruxolitinib, and the BTK inhibitor ibrutinib. As expected, treatment with PHA-408 led to decreased expression of TRAF1 protein. This treatment also resulted in decreased phosphorylation of both STAT3 and ERK. Ruxolitinib treatment only decreased STAT3 activation. In presence of ibrutinib, TRAF1 expression and STAT3 phosphorylation were moderately decreased while ERK phosphorylation was completely abolished (Fig. 4b). As shown in Fig. 4c, PD-L1 mRNA expression was decreased in presence of the three inhibitors. This result was confirmed at the protein level. At the membrane level, PD-L1 expression was strongly reduced 48 h after treatment with either PHA-408 or Ruxolitinib and moderately with ibrutinib (Fig. 4d). These inhibitors also decreased IL-10 expression (Additional file 3: Figure S2). Of note, modulation of PD-L1 expression was no longer observed on wild type CD19_Cre splenocytes treated with inhibitors (Additional file 3: Figure S3). Furthermore, CD40 plus IL-4, BCR, and IL-10R stimulation of CD19_Cre splenocytes induced over-expression of PD-L1, that induction being repressed when cells were treated with these inhibitors (Additional file 3: Figure S4).

**Fig. 5:**
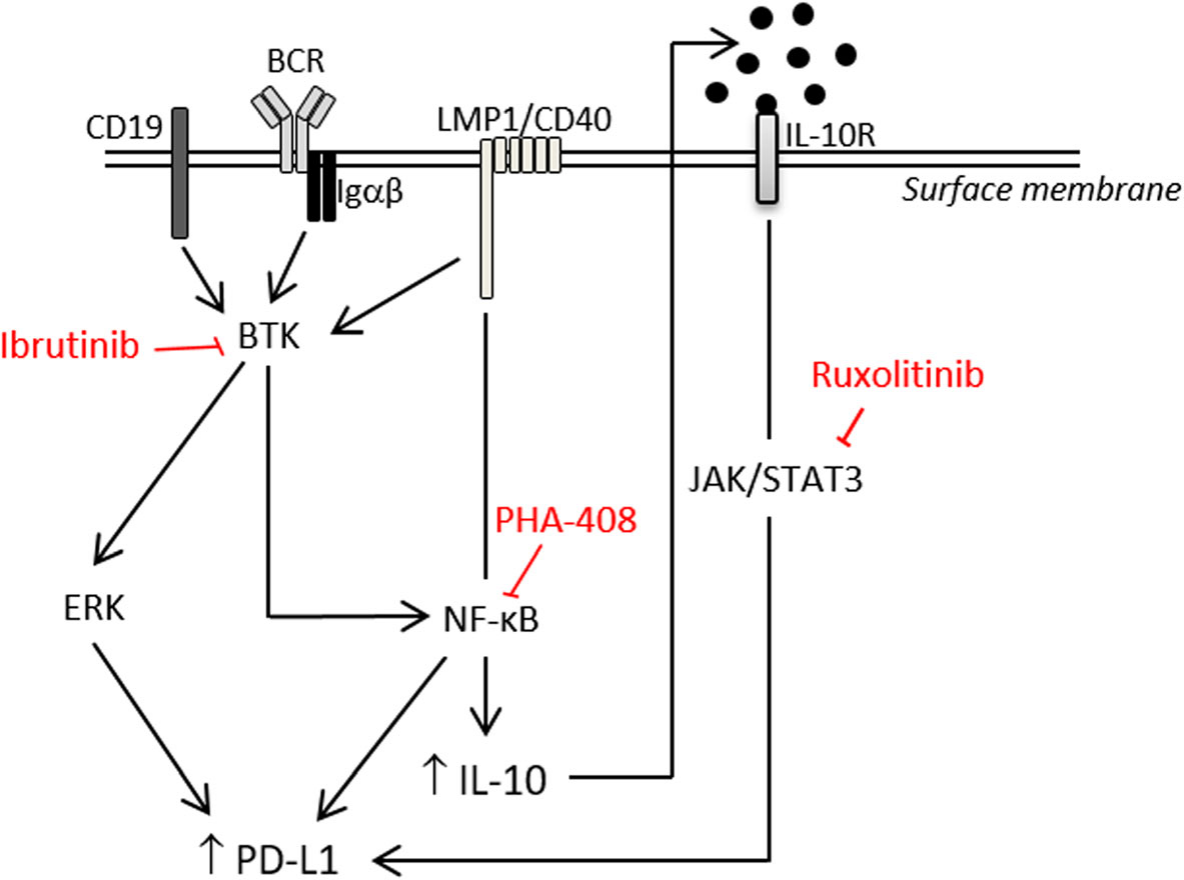
Graphical representation of signaling pathways leading to PD-L1 over-expression in tumor B-cells from LMP1/CD40 mice. CD19, BCR (B-cell receptor), Igαβ, LMP1/CD40, and IL-10R (IL-10 receptor) are located at the surface membrane. CD19 plus BCR/Igαβ complex, and CD40 lead to BTK (*Burton’s tyrosine kinase*) activation which in turn activate ERK and NF-κB that up-regulate PD-L1 expression. Indirectly PD-L1 is also increase by IL-10 by an autocrine loop. PHA-408, inhibitor of NF-κB. Ruxolitinib, inhibitor of JAK1/JAK2. Ibrutinib, inhibitor of BTK

Altogether, these results show that CD40, IL-10 and BCR signaling pathways have complex interplays. As summarized in Fig. 5, the CD40 signaling pathway would be directly responsible for NF-κB activation and would increase the BCR signaling. Both BCR and CD40 signaling would lead to IL-10 secretion that secondary activate the JAK/STAT pathway in an autocrine manner. With NF-κB, both ERK and STAT3 activation would up-regulate expression of PD-L1. Blocking either NF-κB, BCR or STAT3 activation would led to decreased PD-L1 expression. PD-L1 expression was thus under the control of CD40 activation, either directly through NF-κB activation or indirectly through IL-10 induction of BCR sensitization and BTK activation.

## Discussion

Expression of PD-1 and PD-L1 in B-cell lymphomas and effect of immune therapies against the immune checkpoint axe has been recently reviewed [3]. Expression of PD-L1 by tumor cells and effect of anti-PD-1 immune therapy has been clearly demonstrated in Hodgkin’s lymphoma, a tumor which is constantly associated with both NF-κB and STAT3 activation. In DLBCL, expression of PD-L1 is variable but participates to the gene immune escape signature of ABC-DLBCLs [3, 7]. ABC-DLBCL are not only associated with NF-κB activation but may exhibit a chronic active BCR [14] and are sensitive to BTK inhibitors [15]. STAT3 activation mainly found in ABC-DLBCLs and is associated with poor survival [16].

Here, our results show that NF-κB activated B-cell lymphoma of the LMP1/CD40-expressing mouse model exhibited an immune escape gene signature involving expression of PD-L1 and PD-L2, which expression was co-clusterized with IL-10. Over-expression of PD-L1 not only involved NF-κB activation by CD40 but also BTK and JAK/STAT signaling, the latter probably being indirectly regulated via an autocrine loop with participation of IL-10 for example. Indeed, PD-L1 expression could be down-regulated after treated with NF-κB, JAK1/JAK2 and BTK inhibitors. Expression of PD-L1 was very likely to be associated to tumor immune escape as demonstrated for numerous solid cancers such as melanoma of lung cancers [2]. Indeed, in vivo blockade of PD-L1 was able to rapidly repress expansion of these B-cell lymphomas, with concomitant decrease in both B-cell proliferation and B-cell expression of activation markers as well as an increase in T-cell activation. Among these markers are expression of PD-1 on both CD4 and CD8 T-cells. PD-1 is transcriptionally induced in activated T cells [17]. PD-1 expression on activated T-cells allowed subsequent exhaustion and inhibition thank to PD-1/PD-L1 interaction to return to unactivated state [18]. Our results indicate that such T-cell inhibition no longer occurred when PD-L1 was blocked.

## Conclusion

Our results indicate that therapies against the PD-L1/PD-1 axis may work in lymphomas as long as the tumor cells express PD-L1. Our results also suggest that combination of immune therapy targeting the PD-1/PD-L1 axis and TKI specific for the JAK/STAT or the BCR/BTK pathway could be of interest. Even if these chemical inhibitors such as ruxolitinib or ibrutinib could have their own inhibitory effect on actors of the anti-tumor response such as cytotoxic T-cells or macrophages, our results also raise the question of the effect of these molecules on the local reactivation of the immune system.

## Additional files

Additional file 1: **Table S1.** Immune Escape Gene signature adapted to the Affymetrix HG-U133_Plus_2 array. **Table S2.** Immune Escape Gene signature adapted to the HT MG-430 PM Array. (DOCX 32 kb)

Additional file 2: **Table S3.** Normalized expression levels of genes belonging to the immune escape gene signature of splenocytes from LMP1/CD40 and CD19_Cre mice. (DOCX 25 kb)

Additional file 3: **Figure S1.** Absolute number of spleen granulocytes (A) and T-cells (B) in LMP1/CD40-expressing mice after injection of isotype control (Ctrl) or anti-PD-L1 (αPD-L1) antibody. For the PD-L1 treatment, LMP1/CD40-expressing mice were injected avery 4 days for three weeks with 200 µg anti-PD-L1 antibody in On VivoPure Dilution Buffer (clone 10F.9G2; Bio X cell; US). ns, non-significant. **Figure S2.** IL-10 mRNA expression in splenocytes from LMP1/CD40-expressing mice treated with the PHA-408, ruxolitinib and ibrutinib inhibitors. Results are expressed in logarithm of fold change by comparison with the control. **Figure S3.** Analysis of NF-κB/TRAF1, JAK/STAT3, and ERK pathways by western blot of protein extracts from splenocytes of control CD19_Cre mice after 48 h in vitro treatment with the PHA-408, ruxolitinib and ibrutinib inhibitors. GAPDH was used as loading control. **Figure S4.** Analysis of PD-L1 expression by western blot of protein extracts from splenocytes of CD19_Cre mice i) after 48 h in vitro CD40 (R&D Systems), CD40 plus IL-4 (Peprotech), IL-10 (R&D Systems), IgM (Jackson ImmunoResearch) stimulations (lane 2 to 5), and ii) after 24 h in vitro CD40, CD40 plus IL-4, IL-10, IgM stimulations followed by 24 h treatment with the PHA-408, ruxolitinib and ibrutinib inhibitors (lanes 6 to 9). GAPDH was used as loading control. (PPTX 6393 kb)

## Data Availability

The data sets supporting the results of this article are included within the article and its additional files.

## Abbreviations

ABC-DLBCL: Diffuse large B-cell lymphomas with an activated phenotype
BCR: B-cell receptor
BTK Inhibitor (ibrutinib): Bruton’s tyrosine kinase inhibitor
CD19_Cre mice: Mice expressing Cre-recombinase under the control of the B-specific CD19 promoter
EBV: Epstein-Barr virus
LMP1/CD40 protein: Fusion protein containing the amino-terminal part and the transmembrane part of the Epstein-Barr Virus latent membrane protein 1 and the transduction tail of CD40
LMP1/CD40-expressing mice: Mice expressing LMP1/CD40 fusion protein only in B-cell thanks to a stop cassette deletion by Cre-recombinase at the CD19 locus
NF-κB: Nuclear factor kappa B
PD-1: Also known as CD279, Programmed cell death 1
PD-L1: Also known as B7-H1 or CD274, programmed death-ligand 1
PHA-408: An inhibitor of I kappa-B kinase 2
TKI (ruxolitinib): Janus kinase 1/2 tyrosine kinase inhibitor
