# Supplementary material for: Pre-clinical blocking of PD-L1 molecule, which expression is down regulated by NF-κB, JAK1/JAK2 and BTK inhibitors, induces regression of activated B-cell lymphoma
Source: Cell Commun Signal. 2019 Aug 5;17:89. doi: 10.1186/s12964-019-0391-x (PMC6683395; doi:10.1186/s12964-019-0391-x)
Supplement: Supplementary file 3 — Figure S1. Absolute number of spleen granulocytes (A) and T-cells (B) in LMP1/CD40-expressing mice after injection of isotype control (Ctrl) or anti-PD-L1 (αPD-L1) antibody. For the PD-L1 treatment, LMP1/CD40-expressing mice were injected avery 4 days for three weeks with 200 µg anti-PD-L1 antibody in On VivoPure Dilution Buffer (clone 10F.9G2; Bio X cell; US). ns, non-significant. Figure S2. IL-10 mRNA expression in splenocytes from LMP1/CD40-expressing mice treated with the PHA-408, ruxolitinib and ibrutinib inhibitors. Results are expressed in logarithm of fold change by comparison with the control. Figure S3. Analysis of NF-κB/TRAF1, JAK/STAT3, and ERK pathways by western blot of protein extracts from splenocytes of control CD19_Cre mice after 48 h in vitro treatment with the PHA-408, ruxolitinib and ibrutinib inhibitors. GAPDH was used as loading control. Figure S4. Analysis of PD-L1 expression by western blot of protein extracts from splenocytes of CD19_Cre mice i) after 48 h in vitro CD40 (R&D Systems), CD40 plus IL-4 (Peprotech), IL-10 (R&D Systems), IgM (Jackson ImmunoResearch) stimulations (lane 2 to 5), and ii) after 24 h in vitro CD40, CD40 plus IL-4, IL-10, IgM stimulations followed by 24 h treatment with the PHA-408, ruxolitinib and ibrutinib inhibitors (lanes 6 to 9). GAPDH was used as loading control. (PPTX 6393 kb) [file 12964_2019_391_MOESM3_ESM.pptx]

## Slide 1
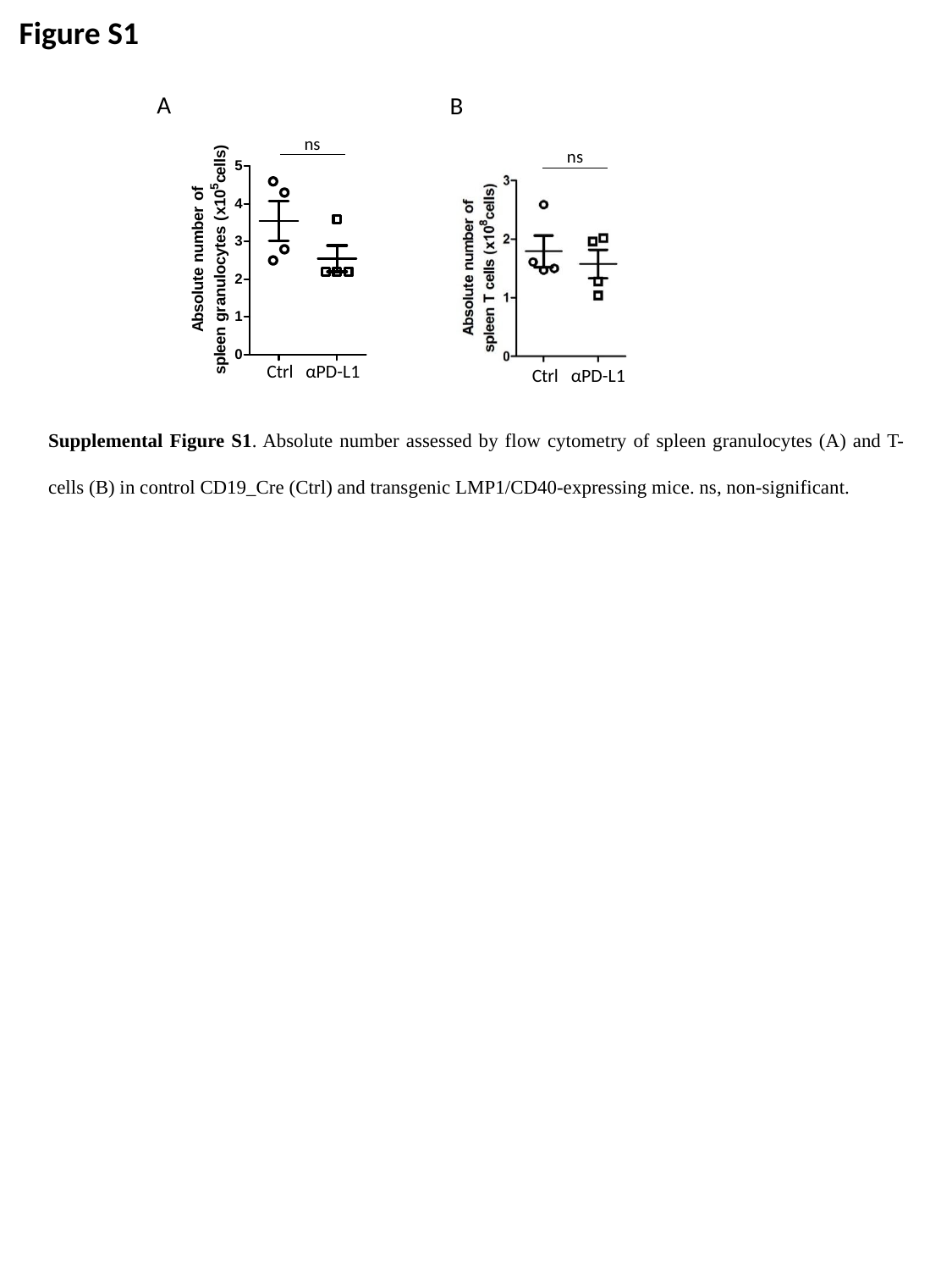

Figure S1
A
B
ns
ns
Ctrl αPD-L1
Ctrl αPD-L1
Supplemental Figure S1. Absolute number assessed by flow cytometry of spleen granulocytes (A) and T-cells (B) in control CD19_Cre (Ctrl) and transgenic LMP1/CD40-expressing mice. ns, non-significant.

## Slide 2
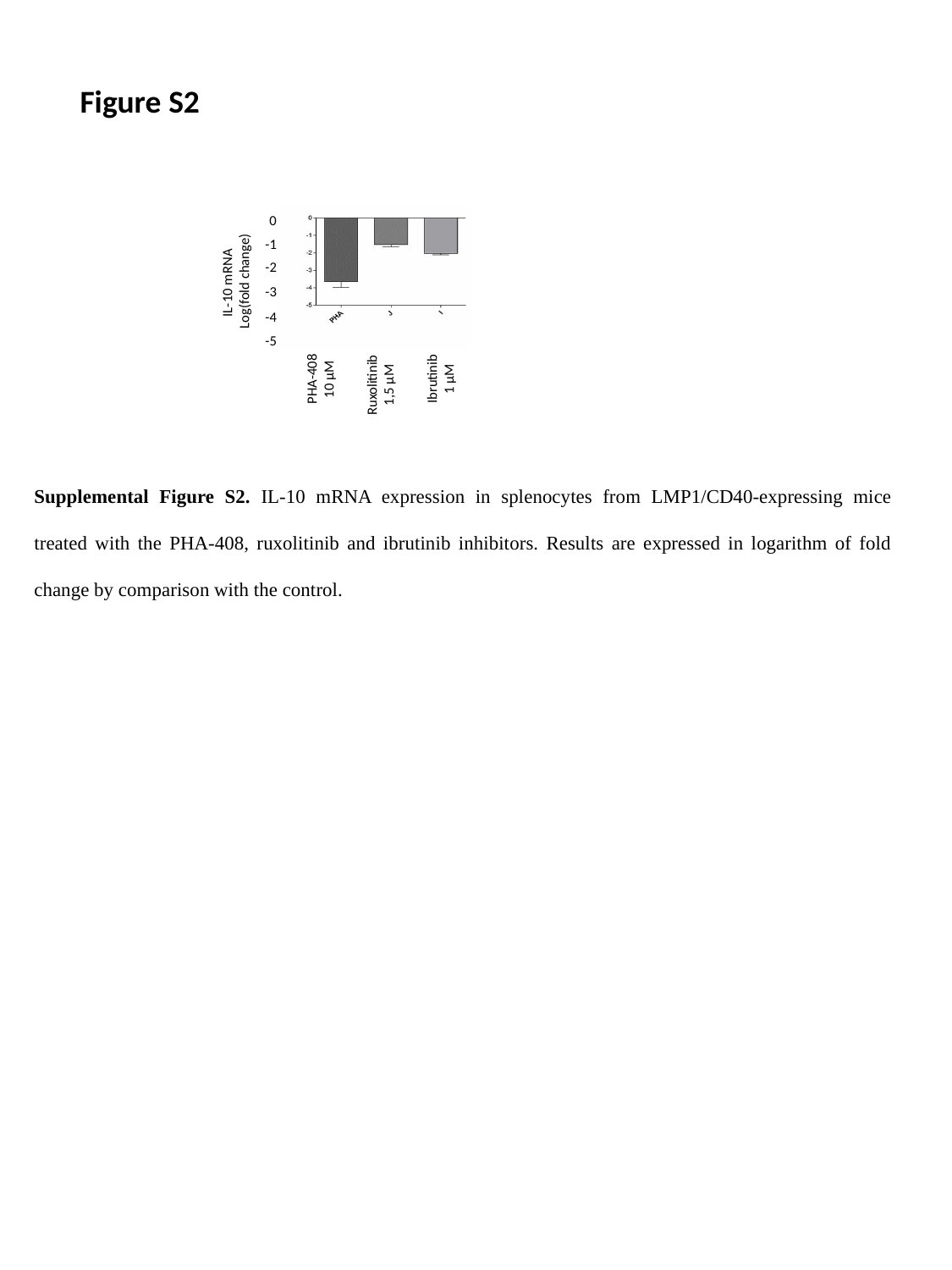

Figure S2
0
-1
-2
IL-10 mRNA
Log(fold change)
-3
-4
-5
Ibrutinib
1 µM
PHA-408
10 µM
Ruxolitinib
1,5 µM
Supplemental Figure S2. IL-10 mRNA expression in splenocytes from LMP1/CD40-expressing mice treated with the PHA-408, ruxolitinib and ibrutinib inhibitors. Results are expressed in logarithm of fold change by comparison with the control.

## Slide 3
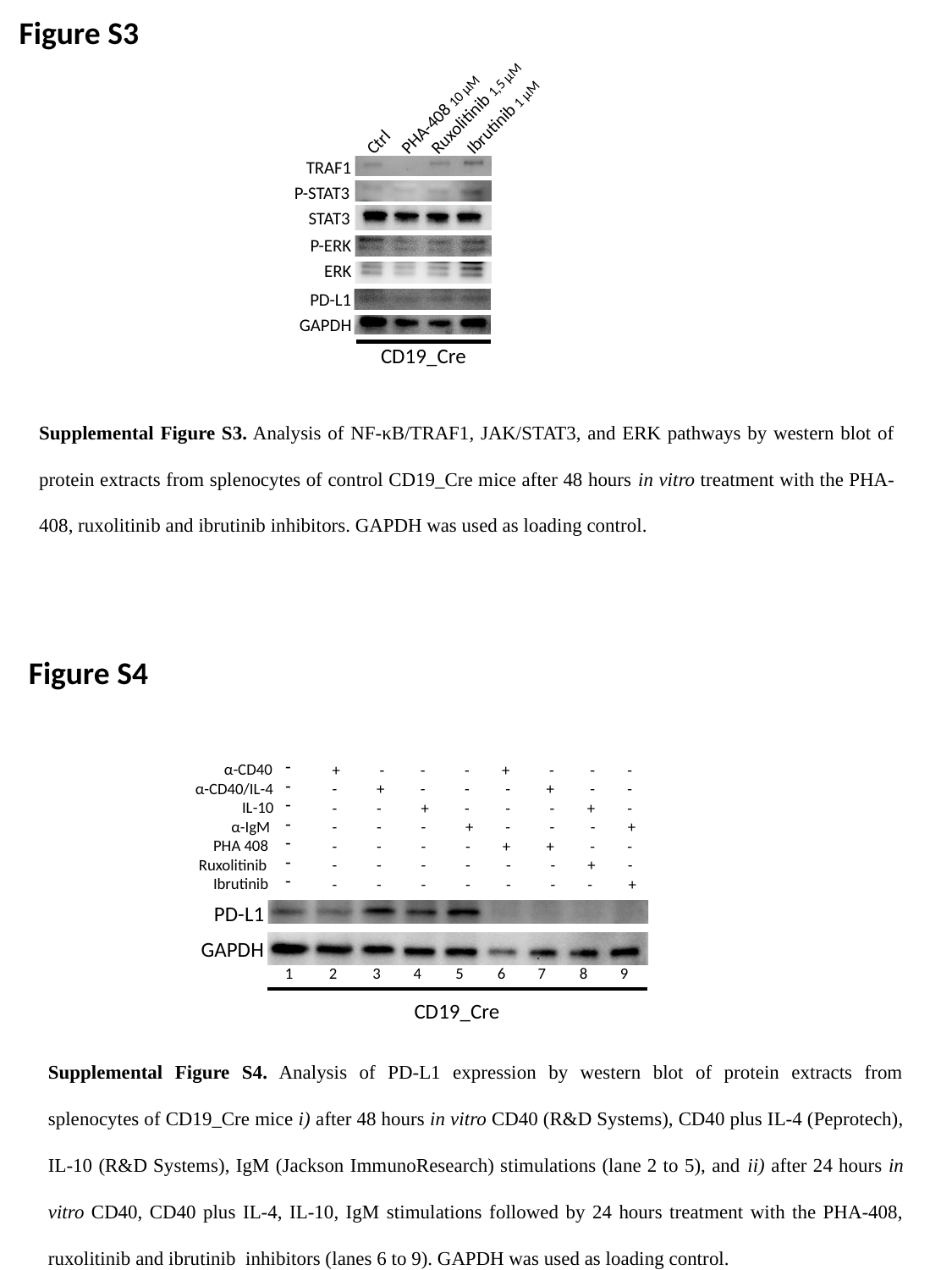

Figure S3
Ruxolitinib 1,5 µM
PHA-408 10 µM
Ibrutinib 1 µM
Ctrl
TRAF1
P-STAT3
STAT3
P-ERK
ERK
PD-L1
GAPDH
CD19_Cre
Supplemental Figure S3. Analysis of NF-κB/TRAF1, JAK/STAT3, and ERK pathways by western blot of protein extracts from splenocytes of control CD19_Cre mice after 48 hours in vitro treatment with the PHA-408, ruxolitinib and ibrutinib inhibitors. GAPDH was used as loading control.
Figure S4
 α-CD40
 α-CD40/IL-4
 IL-10
 α-IgM
 PHA 408
 Ruxolitinib
 Ibrutinib
 + - - - + - - -
 - + - - - + - -
 - - + - - - + -
 - - - + - - - +
 - - - - + + - -
 - - - - - - + -
 - - - - - - - +
PD-L1
GAPDH
1
2
3
4
5
6
7
8
9
CD19_Cre
Supplemental Figure S4. Analysis of PD-L1 expression by western blot of protein extracts from splenocytes of CD19_Cre mice i) after 48 hours in vitro CD40 (R&D Systems), CD40 plus IL-4 (Peprotech), IL-10 (R&D Systems), IgM (Jackson ImmunoResearch) stimulations (lane 2 to 5), and ii) after 24 hours in vitro CD40, CD40 plus IL-4, IL-10, IgM stimulations followed by 24 hours treatment with the PHA-408, ruxolitinib and ibrutinib inhibitors (lanes 6 to 9). GAPDH was used as loading control.
